# Structure-Based Modification of an Anti-neuraminidase Human Antibody Restores Protection Efficacy against the Drifted Influenza Virus

**DOI:** 10.1128/mBio.02315-20

**Published:** 2020-10-06

**Authors:** Haihai Jiang, Weiyu Peng, Jianxun Qi, Yan Chai, Hao Song, Yuhai Bi, Pramila Rijal, Haiyuan Wang, Babayemi O. Oladejo, Jinhua Liu, Yi Shi, George F. Gao, Alain R. Townsend, Yan Wu

**Affiliations:** a College of Veterinary Medicine, China Agricultural University, Beijing, China; b CAS Key Laboratory of Pathogenic Microbiology and Immunology, Institute of Microbiology, Chinese Academy of Sciences (CAS), Beijing, China; c Research Network of Immunity and Health (RNIH), Beijing Institutes of Life Science, Chinese Academy of Sciences, Beijing, China; d Center for Influenza Research and Early-warning (CASCIRE), Chinese Academy of Sciences, Beijing, China; e Center for translational Immunology, Chinese Academy of Medical Science Oxford Institute, Nuffield Department of Medicine, Oxford University, Oxford, United Kingdom; f Department of Pathogen Microbiology, School of Basic Medical Sciences, Capital Medical University, Beijing, China; Virginia Polytechnic Institute and State University

**Keywords:** influenza, neuraminidase, neutralizing antibody, structure-based modification

## Abstract

Here, we investigate a monoclonal antibody, Z2B3, isolated from an H7N9-infected patient, that exhibited cross-reactivity to both N9 (group 2) and a broad range of seasonal and avian N1 (group 1) proteins but lost activity to the N1 with the substitution K432E. This substitution exists in 99.25% of seasonal influenza strains after 2013. The NA-Z2B3 complex structures indicated that Z2B3 binds within the conserved active site of the neuraminidase (NA) protein. A salt bridge between D102 in Z2B3 and K432 in NA plays an important role in binding. Structure-based modification of Z2B3 with D102R in heavy chain reversed the salt bridge and restored the binding and inhibition of N1 with E432. Furthermore, Z2B3-D102R can protect mice from A/Serbia/NS-601/2014 H1N1 virus (NA contains E432) infection while the wild-type Z2B3 antibody shows no protection. This study demonstrates that a broadly reactive and protective antibody to NA can be in principle edited to restore binding and inhibition to recently drifted N1 NA and regain protection against the variant influenza strain.

## Abstract (precis)

The immune system produces antibodies to protect the human body from harmful invaders. The monoclonal antibody (MAb) is one kind of effective antivirals. In this study, we isolated an antibody (Z2B3) from an H7N9 influenza virus-infected child. It shows cross-reactivity to both group 1 (N1) and group 2 (N9) neuraminidases (NAs) but is sensitive to N1 NA with a K432E substitution. Structural analysis of the NA-antibody fragment antigen-binding (Fab) complex provides a clue for antibody modification, and the modified antibody restored binding and inhibition to recently drifted N1 NA and regained protection against the variant influenza strain. This finding suggests that antibodies to NA may be a useful therapy and can be in principle edited to defeat drifted influenza virus.

## INTRODUCTION

Influenza A viruses cause considerable morbidity and mortality annually, posing threats to public health worldwide. In 2009, an emerging swine-origin H1N1 virus reentered the human population and caused the first pandemic in the 21st century. For the first time, novel avian influenza H7N9 virus infected humans in China in 2013, resulting in 15,33 confirmed cases and 607 deaths as of September 2017 (1). The licensed small-molecule anti-influenza drugs, including oseltamivir and zanamivir, are the primary choice for the treatment in the early stage of an H1N1 or H7N9 outbreak. However, drug-resistant virus strains quickly emerge under the pressure of selection (2, 3), and the naturally resistant virus has persisted in viral populations (4, 5). The lasting threat of influenza infection to human populations urgently calls for alternative antiviral countermeasures.

Monoclonal antibodies represent a viable approach for the treatment of influenza. Antibodies targeting hemagglutinin (HA) and neuraminidase (NA), the two important surface glycoproteins of influenza virus, can disrupt viral entry into host cells and block viral egress or prevent virus from budding, respectively. Earlier work defined the structure of NA and NA-fragment antigen-binding (Fab) complexes and demonstrated the protective effects of immune responses to NA (6–8). The importance of NA-based immunity for influenza virus protection has recently been reemphasized (9, 10). Chen et al. isolated a series of NA-targeting protective antibodies elicited in humans following natural H1N1 or H3N2 infection but not vaccinated individuals due to a poorly represented NA antigen in many subunit vaccines (11). An antibody, HCA-2, derived from rabbits immunized with a 9-mer conserved peptide from the NA active site (residues 222–230) can inhibit NA activity across all nine NA subtypes of influenza A virus but was protective only at very high concentrations (12). The murine antibody CD6 was reported to be effective in inhibiting pdmH1N1 virus both *in vitro* and *in vivo* by targeting a unique epitope bridging neighboring NA monomers (13). Another murine antibody, 3c10-3, targeting N9 fully protected mice from lethal challenge with the A/Anhui/1/2013 H7N9 (Anhui/13) strain in both prophylactic and therapeutic treatments (14). Therefore, NA-specific antibodies, especially those targeting conserved epitopes, can serve as therapeutic agents against seasonal and pandemic influenza virus infection.

Information on NA-specific antibody epitopes is important for understanding protective immunity and may contribute to the design of influenza vaccines (15). Recently, an anti-NA monoclonal antibody, Z2B3, isolated from an H7N9-infected donor, was found to cross-inhibit the enzymatic activity of both N1 enzymes from human viruses isolated between 1918 and 2013, some recent avian H5N1 viruses, and N9 enzyme from 2013 H7N9 virus by enzyme-linked lectin assay (ELLA) (16). The loss of activity of Z2B3 on seasonal N1 from viruses isolated after 2013 was localized to an antigenic drift mutation, K432E, at a site previously conserved in seasonal H1N1 viruses (16).

Here, we determined the crystal structures of A/Brevig Mission/1/1918 H1N1 NA (18N1) and A/Anhui/1/2013 H7N9 NA (AH-N9) in complex with the fragment antigen-binding (Fab) portion of Z2B3. We also showed that Z2B3 binds into the conserved active site of NA, that its D102 in heavy chain forms a salt bridge with K432 of NA, and that this accounts for the sensitivity of the antibody to the recent K432E substitution in N1. Structure-based modification of Z2B3 (with substitution of D102R) reestablished the salt bridge in reverse and dramatically restored the binding and inhibition to the N1 with E432 and regained protection against the influenza strain containing E432 in the NA protein.

Our results establish that a broadly protective antibody to NA can bind into the conserved active site of the neuraminidase enzyme but still makes contacts with the rim of the site that can undergo antigenic drift. A loss of binding occurred due to the loss of a specific salt bridge with NA K432 at the edge of the active site, which could be restored in reverse by a targeted mutation in the antibody combining site. This demonstrates that therapeutic antibodies can in principle be edited to keep pace with antigenic drift.

## RESULTS

### Z2B3 exhibits cross-binding to both N1 and N9 proteins.

**FIG 1:**
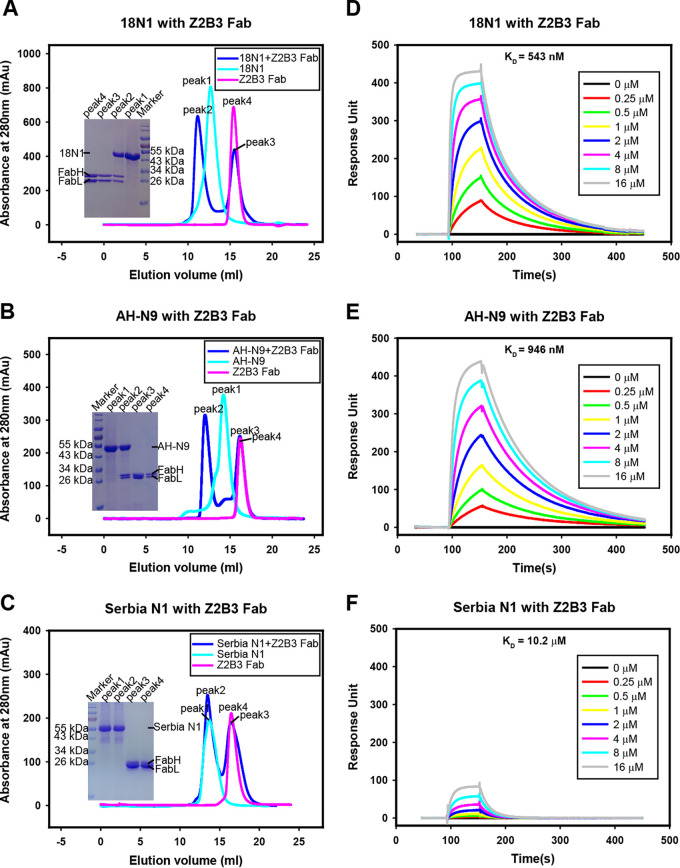
Binding profile of Z2B3 to N1 and N9. (A to C) Gel filtration analysis of Z2B3 Fab survive with 18N1 (A), AH-N9 (B), and Serbia N1 (C). Z2B3 (magenta), NA (cyan), or Z2B3-NA (blue) mixtures were analyzed by a Superdex 200 gel filtration column. The elution fractions were pooled and analyzed by SDS-PAGE. (D to F) An SPR assay characterizing the specific binding between Z2B3 and specific NAs, including 18N1 (D), AH-N9 (E), and Serbia N1 (F). The binding affinity (*K_D_*) values were calculated using a 1:1 Langmuir model produced with BIAcore 3000 analysis software (BIAevaluation version 4.1).

Z2B3 was isolated from a child with acute mild H7N9 infection in 2013 (16). Gel filtration and surface plasmon resonance (SPR) analyses were used to evaluate its binding to recombinant NA proteins produced in the baculovirus expression system. A gel filtration survival assay indicated that Z2B3 Fab can bind to both 18N1 and AH-N9 neuraminidases (Fig. 1A and B) but could not bind to N1 from A/Serbia/NS-601/2014 H1N1 virus containing the K432E substitution (Serbia N1) (Fig. 1C). The binding affinities of Z2B3 to 18N1 and AH-N9 proteins were 543 nM and 946 nM, respectively (Fig. 1D and E), while the binding affinity between Z2B3 and Serbia N1 was 10.2 μM (Fig. 1F), which is much weaker.

### Structural basis of Z2B3 cross-reactivity.

**FIG 2:**
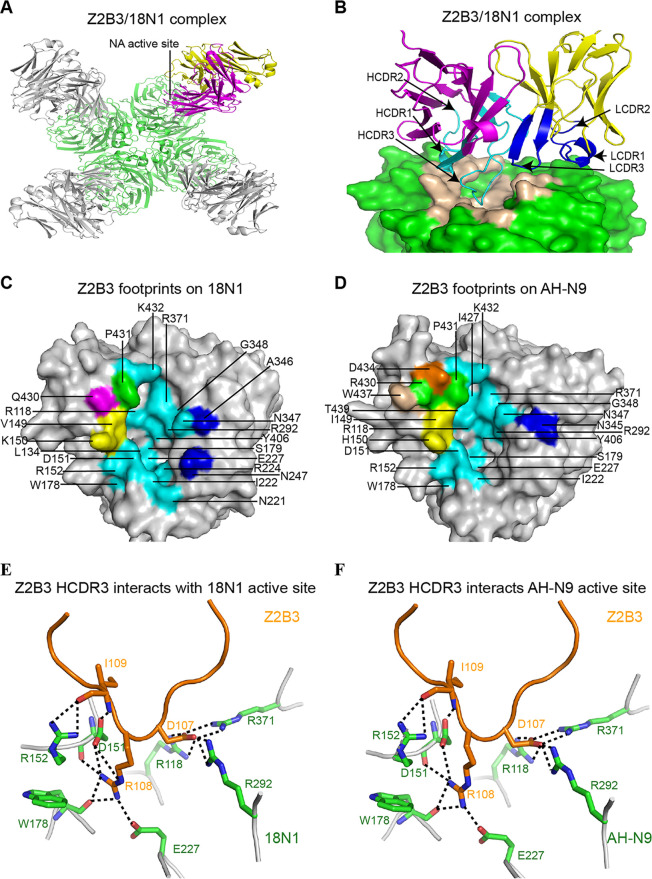
Molecular determinants of Z2B3 with 18N1 and AH-N9. (A) Overall structure of Z2B3/18N1 complex, with NA in green and the heavy chain (HC) and light chain (LC) of one Fab in magenta and in yellow, respectively. Each NA monomer binds to one Z2B3 Fab. (B) The side view of the Z2B3/18N1 complex structure. The HC and LC of Z2B3 are colored in magenta and yellow, respectively. Residues of HC and LC involved in the interactions are colored in cyan and blue, respectively. The epitopes of Z2B3 are colored in wheat. (C and D) The footprints of Z2B3 on 18N1 (C) and AH-N9 (D). NAs are represented in the gray surface. Residues interacting with FR3, HCDR2, and HCDR3 are colored in wheat, magenta, and cyan, respectively. Residues that interact with both HCDR1 and HCDR2 are colored in orange. Residues that interact with both HCDR2 and HCDR3 are colored in yellow. Residues that interact with HCDR1, HCDR2, and HCDR3 are colored in green. Residues interacting with LCDR1 or LCDR3 are colored in blue. Residues contacted less than 4.5 Å are involved. (E and F) The interactions between residues in HCDR3 of Z2B3 and the key residues in the active site of 18N1 (E) or AH-N9 (F). The Z2B3 HCDR3 and NA are shown in orange and green, respectively, in the cartoon representation.

To fully characterize Z2B3 epitopes that contribute to its cross-binding ability, we therefore determined the crystal structures of the Z2B3 Fab complexed with both 18N1 and AH-N9 at 2.5 Å and 2.9 Å, respectively (see Table S1 in the supplemental material). Similar to previously reported NA-antibody complexes (8, 13, 17, 18), one NA tetramer can bind with four Z2B3 Fabs. Alternatively, each NA monomer binds to one Fab. The crystal structures of Z2B3/18N1 and Z2B3/AH-N9 complexes indicated that Z2B3 recognizes the conserved enzyme active site in the globular head domain of N1 and N9 proteins, which partially explained its broad cross-reactivity (Fig. 2A; see Fig. S1A in the supplemental material). Specifically, in the Z2B3/18N1 complex structure, both the heavy chain and the light chain, contribute to binding of the 18N1 protein. The heavy chain of Z2B3 plays a major role in the interaction with 253 atom-to-atom contacts, including frame region 1 (FR1), complementarity determining region 1 of heavy chain (HCDR1), HCDR2, FR3, and HCDR3. The long HCDR3 loop deeply inserts into the NA active site, while the light chain (LCDR1 and LCDR3) contributes only 3 atom-to-atom contacts (Fig. 2B; see Table S2 in the supplemental material). The epitopes cover several secondary elements, including the 150-loop, 340-loop, and the 430-loop. Detailed epitope residues are referred to in Fig. 2C. The NA active site contains 8 catalytic residues (R118, D151, R152, R224, E276, R292, R371, and Y406 in N2 numbering) that are conserved in all subtypes of NA and another 11 framework residues (E119, R156, W178, S179, D/N198, I222, E227, H274, E277, N294, and E425) which support the catalytic residues. The antibody Z2B3 recognizes seven of the eight conserved catalytic residues (except E276) and some of the frame residues (including W178, S179, I222, and E227) as its epitope in 18N1 (Table S2). Z2B3 presents a similar binding mode to AH-N9 (Fig. S1B and Fig. 2D). The heavy chain contributes a total of 279 contacts, while the light chain (only LCDR1 included) contributes 3 atom-to-atom contacts. The majority of conserved residues (including R118, D151, R152, W178, S179, I222, E227, R292, R371, and Y406) within the enzyme active site were within the antibody epitope (see Table S3 in the supplemental material). Residues D107, R108, and I109 are three key residues in the HCDR of Z2B3, which formed salt bridges and hydrogen bonds with the key residues in the active site of both 18N1 and AH-N9 (Fig. 2E and F).

10.1128/mBio.02315-20.1 FIG S1 The complex structure of Z2B3 with AH-N9. (A) Overall structure of Z2B3/AH-N9 complex. NA molecules are shown in green. The heavy chain (HC) and light chain (LC) of one Fab molecule are highlighted in magenta and yellow, respectively. Each NA monomer binds to one Z2B3 Fab. (B) The side view of the Z2B3/AH-N9 complex structure. The AH-N9 molecule is shown in green surface representation, with epitopes in wheat. HC and LC of Z2B3 are displayed the same color as A, with the residues involved in the N9-Z2B3 interactions in cyan and blue, respectively. Download FIG S1, TIF file, 1.9 MB. Copyright © 2020 Jiang et al. 2020 Jiang et al. This content is distributed under the terms of the Creative Commons Attribution 4.0 International license.

10.1128/mBio.02315-20.5 TABLE S1 Data collection and refinement statistics of Z2B3/NA complex. Download Table S1, DOCX file, 0.01 MB. Copyright © 2020 Jiang et al. 2020 Jiang et al. This content is distributed under the terms of the Creative Commons Attribution 4.0 International license.

10.1128/mBio.02315-20.6 TABLE S2 Interactions between Z2B3 and 18N1. Download Table S2, DOCX file, 0.02 MB. Copyright © 2020 Jiang et al. 2020 Jiang et al. This content is distributed under the terms of the Creative Commons Attribution 4.0 International license.

10.1128/mBio.02315-20.7 TABLE S3 Interactions between Z2B3 and AH-N9. Download Table S3, DOCX file, 0.01 MB. Copyright © 2020 Jiang et al. 2020 Jiang et al. This content is distributed under the terms of the Creative Commons Attribution 4.0 International license.

### K432 of N1 is a key residue epitope of Z2B3.

**FIG 3:**
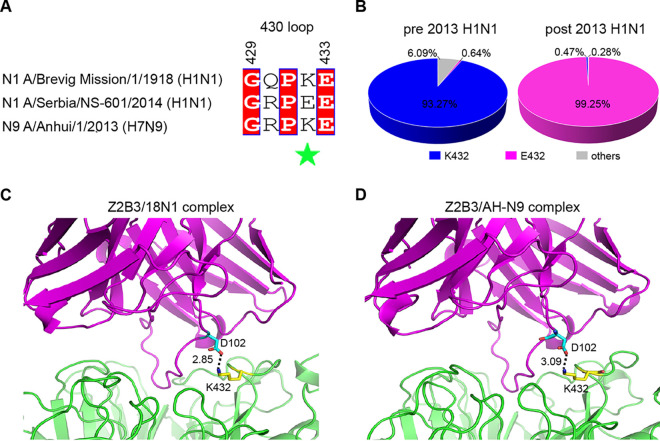
Residue 432 on NA plays a key role in the binding to Z2B3. (A) Sequence alignment of 430-loop among three NAs. Residue 432 is highlighted with a green star. (B) The polymorphisms of residue 432 in N1 from H1N1 seasonal influenza viruses pre- (left) and post (right)-2013 are shown in pie charts. There are 45,999 NA sequences of human H1N1 strains available in the GISAID database (from 1 March 2009 to 31 December 2019), including 13,828 sequences pre-2013 and 32,171 sequences post-2013. The pie charts show the percentage of H1N1 viruses that carry lysine (blue), glutamic acid (pink), or other amino acid residues (gray) at position 432. (C and D) The salt bridge between K432 in 18N1 (B)/AH-N9 (C) and D102 in Z2B3 Fab. The distance is labeled accordingly.

The 4-methylumbelliferyl-*N*-acetylneuraminic acid (MUNANA)-based NA inhibition assay demonstrated that Z2B3 can inhibit the NA activities from H1N1 (A/California/07/2009 and A/Puerto Rico/8/1934), H5N1 (A/Vietnam/1194/2004), and H7N9 (A/Anhui/1/2013) viruses but lost the inhibition activity to an H1N1 virus (A/Serbia/NS-601/2014) (Table 1), which was consistent with previous results (16). This loss of N1 enzyme inhibition was associated with weaker binding to the Serbia N1 protein (Fig. 1C and F). Sequence alignment of Serbia N1, 18N1, and AH-N9 indicated that Z2B3 shared 17 epitope residues on 18N1 and AH-N9 (see Fig. S2 in the supplemental material), while E432 of Serbia N1 is the only residue different from them (N2 numbering) (Fig. 3A). A total of 45,999 NA sequences of human H1N1 strains (from 1 March 2009 to 31 December 2019) were retrieved from the EpiFlu database of the Global Initiative on Sharing All Influenza Data (GISAID). The sequence analysis showed that 99.25% of the seasonal H1N1 viruses circulating in the post-2013 season carry E432 in N1 NA, while only 0.64% of strains contain E432 in the pre-2013 season (Fig. 3B). Residue 432 plays an important role in Z2B3 binding according to the Z2B3/18N1 and Z2B3/AH-N9 complex structures. A salt bridge interaction is formed between the positively charged residue K432 (N2 numbering) of 18N1/AH-N9 and the negatively charged heavy chain residue D102 of Z2B3 (Fig. 3C and D). We hypothesized that the negatively charged E432 in the newly evolved Serbia N1 may hamper the antibody binding because of the electrostatic repulsion. Z2B3 with D102R may enable the binding of N1 with negatively charged E432.

**TABLE 1:** Inhibition of viral replication and NA enzymatic activity of Z2B3 and Z2B3 D102R

Virus	Inhibition of viral replication (μg/ml) by:^*a*^		Inhibition of enzymatic activity (nM) by:^*a*^	
	Z2B3	Z2B3 D102R	Z2B3	Z2B3 D102R
A/California/07/2009 (H1N1)	1.432	10.94	30.41	346
A/Puerto Rico/8/1934 (H1N1)	5.202	20.09	33.07	466.9
A/Serbia/NS-601/2014 (H1N1)	123.7	6.009	>2,000	30.21
A/Vietnam/1194/2004 (H5N1)	7.883	208.3	69.73	628.4
A/Anhui/1/2013 (H7N9)	27.27	305.6	101.3	1,878

a Data were shown as IC_50_ calculated by GraphPad Prism.

10.1128/mBio.02315-20.2 FIG S2 Sequence alignment of epitope residues among Z2B3, NA45, and 1G01. Residues contacted (<4.5 Å) by Z2B3 on 18N1 and AH-N9 are indicated as a magenta triangle and blue rectangle, respectively. Residues contacted (<4.5 Å) by 1G01 on N1 from A/California/04/2009 H1N1 (CA04N1) and NA-45 on mutant N9 from A/Shanghai/02/2013 H7N9 (Sh2 N9 Y169H) are indicated as a yellow circle and orange diamond, respectively. Download FIG S2, TIF file, 2.6 MB. Copyright © 2020 Jiang et al. 2020 Jiang et al. This content is distributed under the terms of the Creative Commons Attribution 4.0 International license.

### Modification of Z2B3 to cope with antigenic drift of virus.

**FIG 4:**
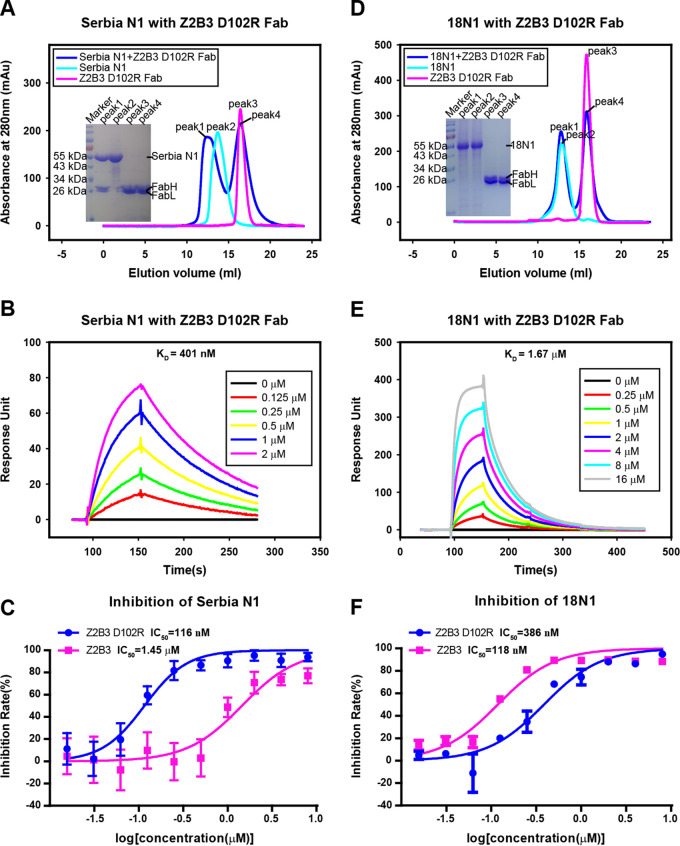
A D102R substitution in Z2B3 can restore the reactivity to Serbia N1. (A and D) Gel filtration survival analysis of Z2B3-D102R Fab with Serbia N1 (A) and 18N1 (D). Z2B3-D102R (magenta), NA (cyan), or Z2B3-D102R-NA (blue) mixtures were analyzed by a Superdex 200 gel filtration column. The elution fractions were pooled and analyzed by SDS-PAGE. (B and E) An SPR assay characterizing the specific binding between Z2B3-D102R and specific NAs, including Serbia N1 (B) and 18N1 (E). The binding affinity (*K_D_*) values were calculated using a 1:1 Langmuir model produced with BIAcore 3000 analysis software (BIAevaluation version 4.1). (C and F) NA inhibition activity of Z2B3 and Z2B3-D102R for Serbia N1 (C) and 18N1 proteins (F).

To test this hypothesis, we modified the antibody Z2B3 with D102R in the heavy chain and then purified the mutant antibody (Z2B3-D102R) in the baculoviral expression system. A gel filtration assay indicated that it could bind to Serbia N1 (Fig. 4A), and the binding affinity measured by SPR was 401 nM (Fig. 4B), which was increased 100-fold compared with the wild-type Z2B3. On the other hand, 18N1 and Z2B3-D102R cannot survive in gel filtration (Fig. 4D), and the binding affinity of Z2B3-D102R to 18N1 (K432) decreased 3.07-fold compared with that of wild-type Z2B3 to 18N1 (Fig. 4E). The complex structure of Serbia N1 and Z2B3-D102R further demonstrated that a salt bridge was formed between the E432 of Serbia N1 and R102 of Z2B3-D102R as expected (see Fig. S3 in the supplemental material).

10.1128/mBio.02315-20.3 FIG S3 The salt bridge between R102 in Z2B3-D102R and E432 in Serbia N1. The structures of Z2B3-D102R (magenta) and Serbia N1 (green) are shown in cartoon representation. Residues R102 (cyan) and E432 (yellow) are indicated as sticks. Download FIG S3, TIF file, 0.9 MB. Copyright © 2020 Jiang et al. 2020 Jiang et al. This content is distributed under the terms of the Creative Commons Attribution 4.0 International license.

The MUNANA-based NA inhibition assay showed that both Z2B3 and Z2B3-D102R inhibit NA activity of the 18N1 protein, with 50% inhibitory concentrations (IC_50_s) of 118 and 386 nM, respectively. In contrast, Z2B3 exhibited poor inhibition to the Serbia N1 protein, with an IC_50_ of 1.45 μM. Z2B3-D102R dramatically restored inhibition activity to Serbia N1, with an IC_50_ of 116 nM (Fig. 4C and F). The NA inhibition ability of Z2B3 and Z2B3-D102R displayed the same trend in the virus level (Table 1).

### Antiviral activity of Z2B3 and the D102R variant *in vitro*.

To further investigate the inhibition of viral replication by Z2B3 and its mutant, we conducted an inhibitory assay on Madin-Darby canine kidney (MDCK) cells. It is not unexpected that the *in vitro* growth of diverse virus strains with K432 in NA, including CA/09, PR/34, and VN/04 viruses, was more effectively inhibited by Z2B3 than by Z2B3-D102R (Table 1). Specifically, viral replications of CA/09, PR/34, and VN/04 viruses were remarkably inhibited by Z2B3 on MDCK cells, with the IC_50_ values of 1.432 μg/ml, 5.202 μg/ml, and 7.883 μg/ml, respectively. The inhibitory effect of Z2B3 against Anhui/13 H7N9 virus was relatively weaker, and the IC_50_ value was 27.27 μg/ml. The inhibition ability of Z2B3 to A/Serbia/NS-601/2014 virus was the weakest, with an IC_50_ of 123.7 μg/ml. In contrast, Z2B3-D102R regained an inhibitory effect against viral growth (IC_50_, 6.009 μg/ml). Moreover, it still showed the inhibition effect to CA/09 and PR/34 viruses, with IC_50_s of 10.94 μg/ml and 20.09 μg/ml, respectively. These data suggested that Z2B3 and Z2B3-D102R were able to inhibit virus yield *in vitro* and implicated their potential for protection *in vivo*.

### Prophylactic and therapeutic efficiencies of Z2B3 antibody and the D102R variant.

**FIG 5:**
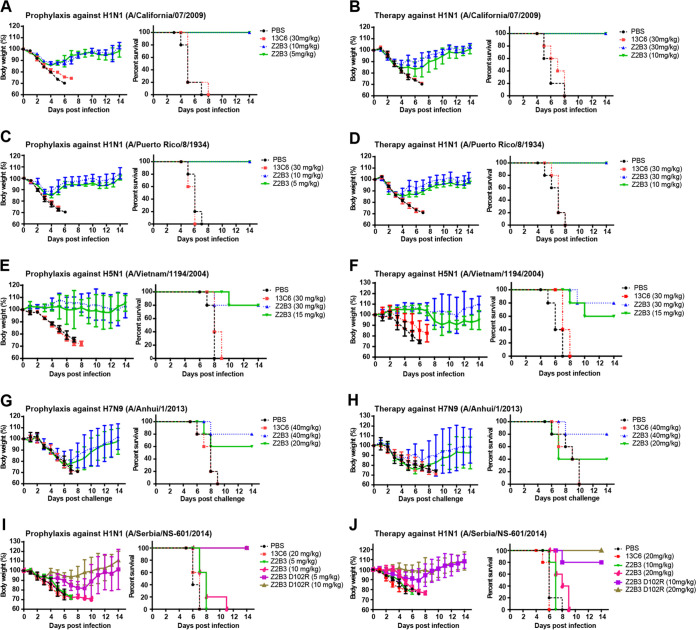
Protection efficacy of Z2B3 or Z2B3-D102R against lethal infection with influenza viruses in mice. (A, C, E, and G) The prophylactic efficacy of Z2B3 against mouse-adapted A/California/07/2009 (H1N1) (A), A/Puerto Rico/8/1934 (H1N1) (C), A/Vietnam/1194/2004 (H5N1) (E), and A/Anhui/1/2013 (H7N9) (G) infection were tested in BALB/c mice (*n* = 5 per group). (B, D, F, and H) The therapeutic efficacy of Z2B3 against mouse-adapted A/California/07/2009 (H1N1) (B), A/Puerto Rico/8/1934 (H1N1) (D), A/Vietnam/1194/2004/2004 (H5N1) (F), and A/Anhui/1/2013 (H7N9) (H) infection were tested in BALB/c or DBA/2 mice (*n* = 5 per group). (I and J) The prophylactic (I) and therapeutic (J) efficacy of Z2B3 or Z2B3-D102R against A/Serbia/NS-601/2014 (H1N1) infection were tested in DBA/2 mice (*n* = 5 per group). Mouse survival rate and body weight were recorded daily. The dose of antibody is labeled accordingly. 13C6 and PBS are set as the negative controls. The survival curves and body weight loss curves were generated using GraphPad Prism 5. Values are mean ± SEM.

Prophylactic and therapeutic studies were performed to explore the protection efficacy of both Z2B3 and Z2B3-D102R against challenge with different viruses *in vivo*. We first tested the prophylaxis and therapy efficacy of Z2B3 against lethal doses of two H1N1 influenza viruses with K432 in NA proteins (mouse-adapted CA/09 and PR/34) in BALB/c mice. Full protection (100%) was observed against lethal infection of these two viruses when mice were administered with 5 or 10 mg/kg of body weight of Z2B3. In contrast, 30 mg/kg of the control monoclonal antibody (MAb; anti-Ebola MAb 13C6) did not protect against weight loss or death (Fig. 5A and C). For the therapeutic efficacy of Z2B3 against CA/09 and PR/34 viruses, mice treated with 10 mg/kg or 30 mg/kg of Z2B3 antibody 12 hours after challenge showed 100% protection (Fig. 5B and D). In contrast, the group treated with irrelative MAb had to be euthanized due to severe weight loss. Moreover, the Z2B3 antibody exhibited prevention of a lethal infection with the avian H5N1 virus A/Vietnam/1194/2004 in BALB/c mice. It was found that a single dose of 15 or 30 mg/kg can protect 80% of mice against death in the prophylactic setting (Fig. 5E). A single dose of 30 mg/kg of Z2B3 can protect 80% of mice against death in the therapeutic group, while 60% of mice treated with 15 mg/kg survived (Fig. 5F). Unexpectedly, Z2B3 exhibited lower prophylactic and protection efficacy to the H7N9 virus (A/Anhui/1/2013), with the survival rate of 80% in the 40 mg/kg group (Fig. 5G and H).

To investigate the protective efficacy of Z2B3-D102R antibody to A/Serbia/NS-601/2014 virus (with E432), we treated the DBA/2 mice with Z2B3-D102R 24 hours prior to intranasal infection in prophylactic study and 12 hours after intranasal infection in the therapeutic experiment. In the prophylactic groups, mice treated with 5 mg/kg or 10 mg/kg of Z2B3-D102R showed 100% protection, while mice treated with the same dose of Z2B3 displayed no protection (Fig. 5I). In the therapeutic groups, mice treated with 10 mg/kg or 20 mg/kg of Z2B3-D102R showed 80% and 100% survival, respectively, while neither counterpart doses of Z2B3 provided any protection (Fig. 5J). These results indicate that D102R substitution could restore the protection by Z2B3 against A/Serbia/NS-601/2014 virus, which was in agreement with the restored inhibition of NA enzymatic activity and viral replication *in vitro*.

## DISCUSSION

NA is an important glycoprotein on the influenza virus surface, which is known as the target of licensed small-molecule inhibitors (oseltamivir, zanamivir, peramivir, and laninamivir). It can also be the target of therapeutic antibodies and an important component of vaccines. Z2B3 is an NA-specific MAb isolated from an H7N9-infected patient, which shows cross-inhibition and protection against examples of seasonal H1N1 viruses isolated in 1934 and 2009, an H5N1 virus from 2004, and an H7N9 virus from 2013 (Table 1 and Fig. 5). However, we observed that the *in vivo* protection efficacy of Z2B3 varied among H1N1, H5N1, and H7N9 subtype influenza viruses. The reason remains to be fully understood, but it could be many factors. The diversity of amino acids in 150-loop, 340-loop, and 430-loop involved in interactions between N1 and N9 may be one of the reasons for the different protective effects. In addition, we cannot rule out that the balance between HA and NA may contribute to the protection difference *in vivo*.

The NA-Z2B3 Fab complex structure revealed that Z2B3 inserted a long HCDR3 loop into the conserved active sites of both N1 and N9 enzymes. However, as predicted by Colman on analyzing the first neuraminidase structures and the first complexes of a murine Fab NC41 with N9 NA, the size of the antigen binding site of the Fab is such that if it occupied the highly conserved active site, it would overlap the rim of the active site, where sequences can vary without interfering with enzyme activity (6, 19). For Z2B3, this led to a loss of activity on H1N1 viruses isolated after 2013 associated with the substitution K432E. This substitution is now fixed in the vast majority of seasonal H1N1 viruses, possibly as a result of immune selection by antibodies similar to Z2B3. The 430-loop in the NA structure is one of the most important antigenic components surrounding the active site and is part of the epitope recognized by Z2B3 through a salt bridge between K432 in NA and D102 in VH. It is of note that a similar salt bridge with K432 was identified in the first structure of a Fab (NC41) with the N9 neuraminidase (6, 7). As Z2B3 might have been useful as a therapeutic agent, we tried restoring its activity by placing a positively charged amino acid (R) to replace the glutamic acid (E) at position 102 in VH. This maneuver restored the salt bridge in reverse, renewed the inhibitory activity of Z2B3 for post-2013 N1 NA, and restored the ability to protect mice from infection with the 2014 H1N1 virus with glutamic acid at position 432. This finding demonstrates the principle that it is possible to “update” therapeutic monoclonal antibodies by strategic mutagenesis to keep pace with antigenic drift.

A similar strategy may work on another glycoprotein (HA) of influenza virus as well. The relatively conserved stem region is probably a better target than the head domain, as it has a much higher adaptive evolution rate. Moreover, some escape mutations on HA can indirectly modulate antibody binding but are not located at the antibody epitope (20). In this case, modification of a single amino acid to restore the effectivity of the antibody to mutant HA may be a challenge.

NA inhibitors (NAIs) are widely used as antiviral drugs for the current clinical treatment and chemoprophylaxis of influenza infections. The emergence of NAI-resistant substitutions is mainly related to the use of NAIs. MAbs may compensate for the reduced effectivity by the resistant substitutions and vice versa. Moreover, the combined therapy with the viral polymerase inhibitor favipiravir and antibody against the HA could provide more eﬀective protection than only the inhibitor or HA antibody therapy (21), suggesting that the combined therapy of NA antibody and NA inhibitor may also be more eﬀective than NA-MAb alone. It may be a good strategy to treat a patient with severe influenza by the combined therapy of NA antibody and NA inhibitor in the future.

Recently, several human MAbs have been described that form contacts within the conserved active site of the NA. Zhu et al. (22) studied a set of MAbs isolated from volunteers vaccinated with an H7N9 subunit vaccine. One of these MAbs, NA45, bound within the N9 active site, but was highly specific to N9 NA, presumably because interactions with N9-specific residues around the rim of the active site contributed significantly to the binding energy of the antibody. NA45 has an epitope that overlaps with that of Z2B3 (17 of 22 contacts defined for N9) (see Fig. S2 and S4 in the supplemental material). In contrast, a group of three antibodies identified by Stadlbauer et al. (23) from plasmablasts isolated from an individual recovering from a seasonal H3N2 infection showed exceptional cross-reactivity for the NAs from group 1 and 2 viruses. The most broadly protective and cross-reactive example, 1G01, has a binding footprint that significantly overlaps with that of Z2B3 (18 of 22 contacts defined for N1) (Fig. S2 and S4). 1G01 also contacts residues on the rim of the site. A detailed comparison indicated that one catalytic residue (E276) and six framework residues (E119, R156, S179, D/N198, E277, and N294) are not included in the footprint of Z2B3 but are in the footprint of 1G01 (Fig. S2 and S4), which may explain the cross-reactivity of 1G01.

10.1128/mBio.02315-20.4 FIG S4 Footprint comparison of Z2B3 with IG01 and NA-45 on N1 and N9. (A and B) The footprints of Z2B3 on 18N1 (A) and AH-N9 (B). (C) The footprints of 1G01 on CA04N1. (D) The footprints of NA-45 on Sh2 N9 Y169H. NAs are represented in gray surface. Residues interacting with heavy chain and light chain are colored in cyan and blue, respectively. Residues interacting with both heavy chain and light chain are colored in orange (contact distance, <4.5 Å). (E and F) The contact sites of Z2B3 and 1G01 or NA-45 are mapped onto N1 NA (PDB: 4B7J) (E) and N9 NA (PDB: 4MWJ) (F). NA is displayed in green surface representation. Z2B3 unique epitopes are colored in yellow, while 1G01 or NA-45 unique epitopes are colored in light blue. The overlapping epitope residues are shown in red. Download FIG S4, TIF file, 2.3 MB. Copyright © 2020 Jiang et al. 2020 Jiang et al. This content is distributed under the terms of the Creative Commons Attribution 4.0 International license.

These observations suggest that while greatly extending the cross-reactivity to group 1 and 2 NAs, antibodies that bind into the highly conserved active site of the NA enzyme are generally likely to overlap the rim of the site that can tolerate amino acid substitutions without the loss of enzyme activity. Inevitably, this idea implies that these antibodies may be able to select resistance mutations and may still be sensitive to antigenic drift.

Our results confirm that exceptionally broadly reactive antibodies to neuraminidase can be selected after influenza infection due in part to the specificity for the highly conserved active site of the enzyme. It is likely that such antibodies arise when individuals are exposed to viruses only distantly related to those that primed them in childhood. Whether engineered immunogens can be designed that will favor the outgrowth of such B cells by vaccination remains to be seen. The more pragmatic approach of deliberately cross-priming with distantly related live-attenuated or subunit influenza vaccines is however possible immediately. Our results emphasize the value of ensuring that influenza vaccines contain an immunogenic neuraminidase component.

## MATERIALS AND METHODS

### Cells and viruses.

Madin-Darby canine kidney (MDCK) cells were cultured in Dulbecco’s modified Eagle’s medium (DMEM) (Gibco) with 10% fetal bovine serum (FBS) (Gibco) in 37°C with a humidified atmosphere of 5% CO_2_. Spodoptera frugiperda (Sf9) cells and Trichoplusia ni (High Five) (Invitrogen) cells were cultured in serum-free Insect-XPRESS (Lonza, Basel, Switzerland) media in a 27°C shaking incubator. Mouse-adapted A/California/07/2009 (H1N1), A/Puerto Rico/8/1934 (H1N1), A/Vietnam/1194/2004 (H5N1), and A/Anhui/1/2013 (H7N9) were propagated in specific-pathogen free (SPF) embryonated eggs (Vital River Laboratories, Beijing, China), while A/Serbia/NS-601/2014 (H1N1) was propagated in MDCK cells. The virus titers were expressed as 50% tissue culture infective dose (TCID_50_) and determined in MDCK cells.

### Expression and purification of soluble NA proteins.

The NAs (residues 83 to 468) of the A/Brevig Mission/1/1918 (H1N1), A/Serbia/NS-601/2014 (H1N1), and A/Anhui/1/2013 (H7N9) strains were cloned, expressed, and purified as described previously (24–30). Briefly, the NA segments were codon-optimized, commercially synthesized (Genewiz, Suzhou, China), and cloned into pFastBac1 (Invitrogen) in-frame with a GP67 signal peptide, a His_6_ tag, a tetramerization sequence, and a thrombin cleavage site at the N terminus. Recombinant baculovirus was prepared in Sf9 cells. High Five cells were infected with recombinant baculovirus for expression, and the supernatants were collected at 48 hours postinfection. The secreted soluble NA was recovered from filtered supernatant by using a 5-ml HisTrap high-performance (HP) column (GE Healthcare), and the elution fraction was dialyzed. For analytic gel filtration, surface plasmon resonance (SPR), and MUNANA-based neuraminidase inhibition assays, a NA tetramer with His tag attached was concentrated and then purified by gel filtration chromatography using a Superdex-200 Increase 10/300 GL column (GE Healthcare) with a buffer containing 20 mM Tris-HCl (pH 8.0) and 150 mM NaCl. For crystal structure determination, the NA tetramer was subjected to thrombin treatment (3 units per mg NA; Sigma) overnight at 4°C to remove the His tag from the NA ectodomain. The digested NA protein was purified by subsequent gel filtration chromatography using a Superdex-200 Increase 10/300 GL column (GE Healthcare) with a buffer containing 20 mM Tris-HCl (pH 8.0) and 150 mM NaCl. High-purity NA fractions were pooled and concentrated using a membrane concentrator with a molecular mass cutoff of 30 kDa (Millipore).

### Antibody expression and preparation of Fab fragments.

Full-length cDNA of the heavy chain and light chain of the antibody were codon-optimized, commercially synthesized (Genewiz, Suzhou, China), and cloned into pFastBac-Dual (Invitrogen). The IgG proteins were expressed by High Five cells and then purified by protein A and HiLoad 16/600 Superdex 200 columns (GE Healthcare). The purified IgG was used for inhibiting assays and *in vivo* animal study. Fab was used for binding analysis and crystal structure determination. To prepare Fab fragments, IgG was digested with papain using a Pierce Fab preparation kit (Thermo) at an antibody to papain ratio of 160:1 (wt/wt) at 37°C for 6 to 8 h. Fab was separated from the digestion mixture by using a protein A column and purified to homogeneity by subsequent size exclusion gel filtration chromatography (HiLoad 16/600 Superdex 200 pg; GE Healthcare).

### Binding kinetics.

The interactions between Z2B3 or Z2B3-D102R with purified 18N1, Serbia N1, or AH-N9 were measured by SPR using a BIAcore 3000 system (GE Healthcare) at 25°C. The buffers for all proteins used for kinetic analysis were exchanged to phosphate-buffered saline with Tween 20 (PBST) (pH 7.4) consisting of 137 mM NaCl, 2.7 mM KCl, 4.3 mM Na_2_HPO_4_, 1.4 mM KH_2_PO_4_, and 0.005% (vol/vol) Tween 20. Soluble tetramer NA proteins at 20 μg/ml in 10 mM sodium acetate (pH 5.5) were immobilized onto a CM5 chip with the amino coupling method to obtain the required immobilization level of 1,000 response units (RUs). Two-fold serial dilutions of Z2B3 Fab or Z2B3-D102R Fab were prepared and sequentially injected at a flow rate of 30 μl/min for 60 s, followed a dissociation step for 300 s. The association and disassociation curves were recorded, and the background binding was subtracted. The kinetic binding was analyzed, and the affinity constants were calculated using BIAevaluation 4.1 software.

### Analytical gel filtration and purification of NA/Fab complex.

The purified NA protein was mixed with Z2B3 or Z2B3-D102R Fab at a 4.5:1 molar ratio and incubated on ice for 1 hour. The mixture was then loaded onto a Superdex-200 Increase 10/300 GL column (GE Healthcare) in 20 mM Tris-HCl (pH 8.0) and 50 mM NaCl. The elution fractions were pooled and analyzed on a 15% sodium dodecyl sulfate-polyacrylamide gel electrophoresis (SDS-PAGE) gel.

### Crystallization, data collection, and structure determination.

The NA/Fab complex was purified by gel filtration and then concentrated to 10 mg/ml. Crystal screening was performed using a sitting-drop vapor diffusion method at 18°C. The Z2B3 Fab-18N1 complex crystals were grown in the reservoir solution comprising of 4% Tacsimate (pH 7.0) and 20% PEG3350. Z2B3 Fab-AH-N9 complex crystals were obtained under the condition of 0.1 M HEPES (pH 7.5), and 12% PEG3350. Z2B3-D102R Fab-Serbia N1 complex crystals were obtained in a solution consisting of 0.1 M magnesium acetate, 0.1 M 2-(*N*-morpholino)ethanesulfonic acid (MES) (pH 6.5), and 10% (wt/vol) PEG10000. Diffraction data were collected at Shanghai Synchrotron Radiation Facility (SSRF) BL19U1. For data collection, crystals were cryoprotected by soaking in reservoir solution supplemented with 20% glycerol before flash cooling in liquid nitrogen. All the data sets were processed with HKL2000 software (31). Molecular replacement by using the Phaser program (32) was employed with previously reported structures of N1 (PDB code: 3BEQ), N9 (PDB code: 4MWJ), and Fab (PDB code: 5W0D). Coot and Phenix were used for model building and structure refinement (33). The stereochemical qualities of the final models were validated with MolProbity (34). Data collection and refinement statistics are summarized in Table S1.

### MUNANA-based neuraminidase inhibition assay.

For MUNANA-based neuraminidase inhibition assays, a series of 2-fold-diluted antibody was mixed with tetrameric NA proteins or viruses and incubated at 37°C for 30 min prior to the addition of the 167 μM 4-MUNANA (4-methylumbelliferyl-*N*-acetylneuraminic acid) as a fluorescent NA substrate, and the fluorescence was quantified on a microplate reader (SpectrMax M5; Molecular Devices) at an excitation wavelength of 355 nm and an emission wavelength of 460 nm for 30 min. Single time points were chosen where the positive control produced a fluorescence signal of approximately 1,000. All assays were done in triplicates, and the IC_50_ for the antibody was calculated using GraphPad Prism software.

### Viral replication inhibiting assay.

MDCK cells were seeded in 96-well plates 1 day before the experiment. Cells were washed 3 times with PBS and incubated in 100 μl per well of DMEM without FBS. Two-fold serially diluted MAbs (Z2B3 or Z2B3-D102R) were mixed with an equal volume of 100 TCID_50_ virus for 1 h at 37°C. The mixture was then transferred to MDCK cells in 96-well plates for an additional 20-h incubation at 37°C. An irrelevant antibody (anti-Ebola MAb 13C6) was used as a negative control. Both the virus-only controls and cell-only controls were included in each plate. The cell monolayers were then washed with PBS, fixed with cold acetone for 10 min, and blocked for 60 min with 5% nonfat-dried milk. The viral antigen was detected by enzyme-linked immunosorbent assay (ELISA) using an antinucleoprotein (NP) antibody (Medix Biochemica) and horseradish peroxidase-conjugated goat anti-mouse IgG (Santa Cruz) as the primary antibody and secondary antibody, respectively. After being developed with 3,3′,5,5″-tetramethylbenzydine (TMB; Sigma) substrate for 10 min at room temperature, 2 M HCl was applied to stop the reaction. Absorbance at 450 nm was measured and recorded. The 50% inhibitory concentration (IC_50_) was generated using Prism software (GraphPad).

### Animal study.

Animal experiments were performed in accordance with the protocol approved by the Laboratory Animal Welfare and Ethics Committee in Chinese Academy of Sciences. In the prophylactic study against A/Puerto Rico/8/1934 (H1N1) and mouse-adapted A/California/07/2009 (H1N1) viruses, groups of 5 female BALB/c mice (Vital River Laboratories, Beijing, China) aged 6 weeks received doses of 5 and 10 mg/kg of intraperitoneal (i.p.) Z2B3 antibody. A dose of 30 mg/kg anti-Ebola MAb 13C6 or an equivalent volume of PBS was i.p. administered as controls. After 24 hours of treatment, mice were anaesthetized with isoflurane and then intranasally inoculated with 50 μl PBS containing a 1,000 50% lethal dose (LD_50_) of H1N1 viruses. In the therapeutic studies, mice (*n* = 5) received doses of 10 or 30 mg/kg of i.p. Z2B3 antibody 12 hours after intranasal inoculation of 1,000 LD_50_ of H1N1 viruses.

In the prophylactic study against A/Vietnam/1194/2004 (H5N1) virus, 6-week-old female BALB/c mice (Vital River Laboratories, Beijing, China) were passively immunized with 15 or 30 mg/kg of Z2B3 antibody in a final volume of 200 μl. Control mice received 30 mg/kg anti-Ebola MAb 13C6 or PBS. After 24 hours of treatment, the mice were anesthetized and then intranasally infected with 80 LD_50_ of H5N1 virus. In a therapeutic setting, mice received doses of i.p. Z2B3 12 hours after intranasal inoculation with 80 LD_50_ of H5N1 virus (in a 50-μl inoculum).

Five-week-old female DBA/2 mice (*n* = 5 per group) were used for examining the prophylactic or therapeutic efficacy of Z2B3 against A/Anhui/1/2013 (H7N9) infection. Doses of antibody (20 or 40 mg/kg) or control MAb were administered 24 hours prior to or 12 hours after intranasal infection (30 LD_50_).

In the prophylaxis experiments against A/Serbia/NS-601/2014 (H1N1) virus, groups of 5-week-old female DBA/2 mice were given 5 or 10 mg/kg of i.p. Z2B3 or Z2B3-D102R antibody after acclimatization for 3 days. A total of 1,000 LD_50_ of the virus was inoculated via the intranasal route under anesthesia 24 hours after MAb administration. In the therapeutic experiments, each group received doses of Z2B3 or Z2B3-D102R antibody (10 or 20 mg/kg) 12 hours after intranasal infection with 1,000 LD_50_ of A/Serbia/NS-601/2014 (H1N1). Mice in control groups were given 20 mg/kg of 13C6 as an antibody isotype control or an equivalent volume of PBS.

In all groups, body weights and survival rates of all mice were monitored and recorded for 14 days. Mice that displayed 30% or more loss of their initial body weight were subsequently euthanized.

### Data availability.

The data that support the findings of this study are available from the corresponding authors upon request. The crystal structures of Z2B3-18N1, Z2B3-N9, and Z2B3-D102R-Serbia N1 have been deposited in the Protein Data Bank under accession codes 6LXI, 6LXJ, and 6LXK, respectively.
