# Supplementary figures and images for: Structure-Based Modification of an Anti-neuraminidase Human Antibody Restores Protection Efficacy against the Drifted Influenza Virus
Source: mBio. 2020 Oct 6;11(5):e02315-20. doi: 10.1128/mBio.02315-20 (PMC7542365; doi:10.1128/mBio.02315-20)

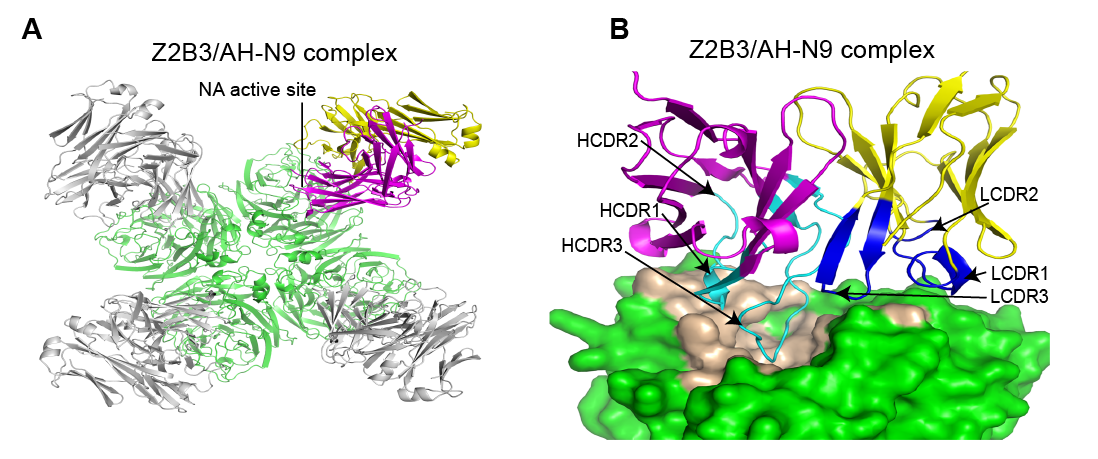

Supplement: FIG S1 [file mBio.02315-20-sf001.tif]

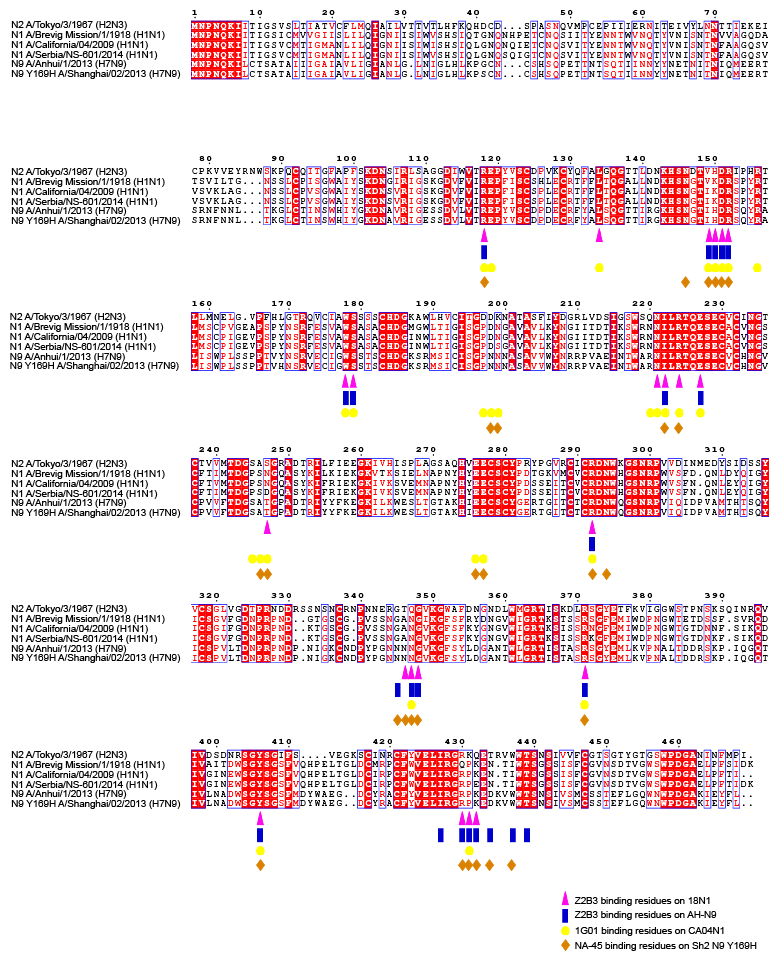

Supplement: FIG S2 [file mBio.02315-20-sf002.tif]

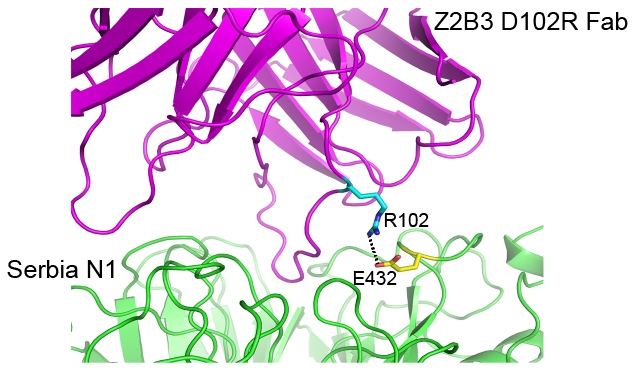

Supplement: FIG S3 [file mBio.02315-20-sf003.tif]

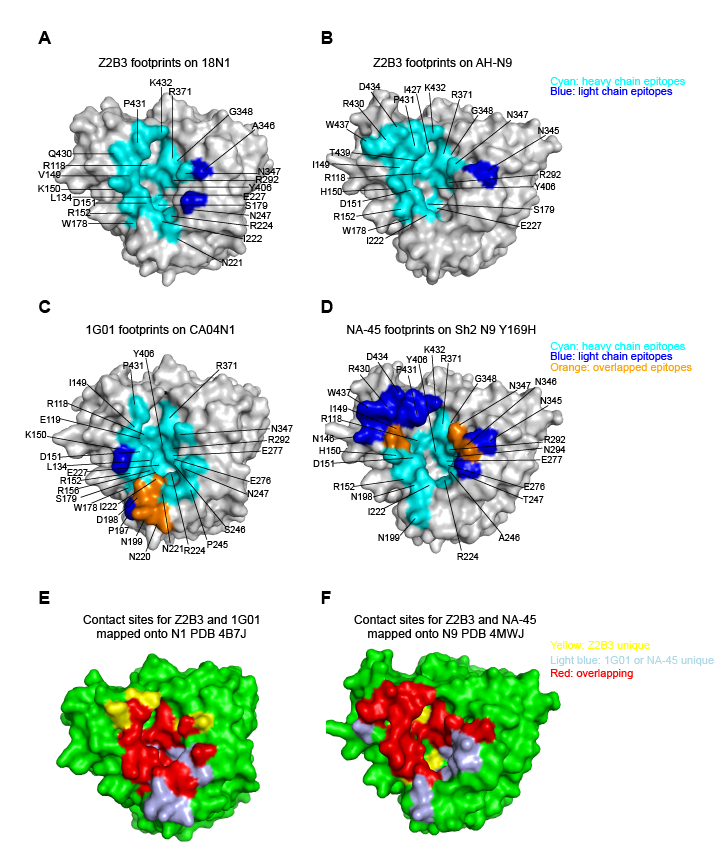

Supplement: FIG S4 [file mBio.02315-20-sf004.tif]
