# Supplementary material for: Structure-Based Modification of an Anti-neuraminidase Human Antibody Restores Protection Efficacy against the Drifted Influenza Virus
Source: mBio. 2020 Oct 6;11(5):e02315-20. doi: 10.1128/mBio.02315-20 (PMC7542365; doi:10.1128/mBio.02315-20)
Supplement: TABLE S1 [file mBio.02315-20-st001.docx]

**Table S1. Data collection and refinement statistics of Z2B3/NA complex**

|  | Z2B3/18N1 | Z2B3/AH-N9 | Z2B3 D102R/Serbia N1 |
| --- | --- | --- | --- |
| Data collection |  |  |  |
| Space group | P42_1_2 | C222 | P3_2_2_1_ |
| Cell dimensions |  |  |  |
| *a,b,c* (Å) | 163.130, 163.130, 190.757 | 201.307, 207.514, 207.702 | 214.441, 214.441, 168.682 |
| α,β,γ (°) | 90.000, 90.000, 90.000 | 90.000, 90.000, 90.000 | 90.000, 90.000,  120.000 |
| Resolution (Å) | 50.00-2.50 (2.59-2.50) | 50.00-2.90 (3.00-2.90) | 50.00-3.60 (3.73-3.60) |
| *I/σ(I)* | 18.0 (3.0) | 16.7 (2.7) | 6.4 (2.3) |
| Completeness (%) | 100.0 (100.0) | 99.4 (99.1) | 100.0 (100.0) |
| Redundancy | 11.1 (11.4) | 6.7 (6.4) | 8.3 (7.9) |
| Unique reflections | 89316 | 95195 | 52001 |
| Refinement |  |  |  |
| Resolution (Å) | 50.00-2.50 (2.59-2.50) | 50.00-2.90 (3.00-2.90) | 50.00-3.60 (3.73-3.60) |
| No. of Reflections | 88386 | 94896 | 51355 |
| R-work/ R-free | 18.65/21.44 | 19.32/24.12 | 21.47/24.88 |
| No. of atoms |  |  |  |
| Protein | 12297 | 24832 | 21987 |
| Ligand/ion | 124 | 691 | 164 |
| Water | 565 |  |  |
| B-factors |  |  |  |
| Protein | 35.700 | 39.876 | 97.211 |
| Ligand/ion | 30.002 | 75.902 | 63.751 |
| Water | 34.638 |  |  |
| R.m.s.d |  |  |  |
| Bond length (Å) | 0.012 | 0.012 | 0.003 |
| Bond angles (°) | 1.032 | 1.223 | 0.634 |
| Ramachandran Plot (%) |  |  |  |
| Favoured | 96.82 | 94.92 | 99.26 |
| Allowed | 3.18 | 4.92 | 0.74 |
| Outliers | 0.00 | 0.16 | 0.00 |
