# Supplementary material for: Structure-Based Modification of an Anti-neuraminidase Human Antibody Restores Protection Efficacy against the Drifted Influenza Virus
Source: mBio. 2020 Oct 6;11(5):e02315-20. doi: 10.1128/mBio.02315-20 (PMC7542365; doi:10.1128/mBio.02315-20)
Supplement: TABLE S2 [file mBio.02315-20-st002.docx]

**Table S2. Interactions between Z2B3 and 18N1**

|  | Antibody | Contacts*^a^* | 18N1*^b^* | Total contacts |
| --- | --- | --- | --- | --- |
| Heavy Chain | K19 | 8 | MAN*^c^* | 253 |
|  | S31 | 4 | P431 |  |
|  | P53 | 3 | FUC*^c^* |  |
|  | V54 | 2, 1, 6, 3, 10 | V149, K150, Q430, P431, FUC*^c^* |  |
|  | Y55 | 2, 2, 5, 1 | V149, K150, P431, FUC*^c^* |  |
|  | G56 | 1 | FUC*^c^* |  |
|  | A72 | 6 | NAG*^c^* |  |
|  | D73 | 19, 10 | NAG*^c^*, BMA*^c^* |  |
|  | E74 | 1, 6, 3 | NAG*^c^*, FUC*^c^*, NAG*^c^* |  |
|  | S75 | 8 | NAG*^c^* |  |
|  | Y80 | 3, 9 | BMA*^c^*, MAN*^c^* |  |
|  | D102 | 5 | K432 |  |
|  | T103 | 1 | P431 |  |
|  | P104 | 3 | N347 |  |
|  | M105 | 2, 11, 5, 4 | N347, R371, P431, K432 |  |
|  | D107 | 6, 9, 6, 1, 9,  11 | R118, D151, R292, G348, R371, Y406 |  |
|  | R108 | 1, 22, 4, 8, 5,  1, 5 | L134, D151, R152, W178, S179, R224, E227 |  |
|  | I109 | 1, 2, 7, 7 | V149, K150, D151, R152 |  |
|  | I110 | 1, 3 | N221, I222 |  |
| Light Chain | Y34 | 1 | A346 | 3 |
|  | R95 | 1 | N247 |  |
|  | S97 | 1 | N247 |  |

*^a^* Contacts represent the number of atom-to-atom interactions between the Z2B3 residues and the 18N1 residues using 4.5 Å as the distance cutoff; *^b^* N2 numbering; *^c^* N-146 linked sugar
