# Supplementary material for: Structure-Based Modification of an Anti-neuraminidase Human Antibody Restores Protection Efficacy against the Drifted Influenza Virus
Source: mBio. 2020 Oct 6;11(5):e02315-20. doi: 10.1128/mBio.02315-20 (PMC7542365; doi:10.1128/mBio.02315-20)
Supplement: TABLE S3 [file mBio.02315-20-st003.docx]

**Table S3. Interactions between Z2B3 and AH-N9**

|  | Antibody | Contacts**^a^** | AH-N9^b^ | Total  contacts |
| --- | --- | --- | --- | --- |
| Heavy Chain | K19 | 17 | MAN*^c^* | 279 |
|  | S30 | 2, 5 | R430, D434 |  |
|  | S31 | 8, 9 | P431, D434 |  |
|  | V54 | 4, 8, 4, 3 | I149, R430, P431, D434 |  |
|  | Y55 | 5, 6, 1 | I149, H150, P431 |  |
|  | A72 | 5 | NAG*^c^* |  |
|  | D73 | 13, 9 | NAG*^c^*, BMA*^c^* |  |
|  | E74 | 6, 9, 2 | R430, W437, NAG*^c^* |  |
|  | S75 | 2 | NAG*^c^* |  |
|  | Y80 | 2, 15 | BMA*^c^*, MAN*^c^* |  |
|  | D102 | 5 | K432 |  |
|  | T103 | 2, 1 | P431, K432 |  |
|  | P104 | 1 | N347 |  |
|  | M105 | 2, 6, 11, 3, 1,  4, 1 | R118, N347, R371, I427, R430, P431, T439 |  |
|  | V106 | 1 | N347 |  |
|  | D107 | 7, 8, 5, 1, 9,  11 | R118, D151, R292, G348, R371, Y406 |  |
|  | R108 | 21, 5, 8, 5, 5 | D151, R152, W178, S179, E227 |  |
|  | I109 | 2, 4, 7, 7 | I149, H150, D151, R152 |  |
|  | I110 | 1 | I222 |  |
| Light Chain | Y32 | 3 | N345 | 3 |

*^a^* Contacts represent the number of atom-to-atom interactions between the Z2B3 residues and the 18N1 residues using 4.5 Å as the distance cutoff; *^b^* N2 numbering; *^c^* N-146 linked sugar
